# Salidroside Improves Homocysteine-Induced Endothelial Dysfunction by Reducing Oxidative Stress

**DOI:** 10.1155/2013/679635

**Published:** 2013-03-26

**Authors:** Sin Bond Leung, Huina Zhang, Chi Wai Lau, Yu Huang, Zhixiu Lin

**Affiliations:** ^1^School of Chinese Medicine, The Chinese University of Hong Kong, Hong Kong; ^2^Institute of Vascular Medicine, Li Ka Shing Institute of Health Sciences and School of Biomedical Sciences, The Chinese University of Hong Kong, Hong Kong

## Abstract

Hyperhomocysteinemia is associated with an increased risk for cardiovascular diseases through increased oxidative stress. Salidroside is an active ingredient of the root of *Rhodiola rosea* with documented antioxidative, antihypoxia and neuroprotective properties. However, the vascular benefits of salidroside against endothelial dysfunction have yet to be explored. The present study, therefore, aimed to investigate the protective effect of salidroside on homocysteine-induced endothelial dysfunction. Functional studies on the rat aortas were performed to delineate the vascular effect of salidroside. DHE imaging was used to evaluate the reactive oxygen species (ROS) level in aortic wall and endothelial cells. Western blotting was performed to assess the protein expression associated with oxidative stress and nitric oxide (NO) bioavailability. Exposure to homocysteine attenuated endothelium-dependent relaxations in rat aortas while salidroside pretreatment rescued it. Salidroside inhibited homocystein-induced elevation in the NOX2 expression and ROS overproduction in both aortas and cultured endothelial cells and increased phosphorylation of eNOS which was diminished by homocysteine. The present study shows that salidroside is effective in preserving the NO bioavailability and thus protects against homocysteine-induced impairment of endothelium-dependent relaxations, largely through inhibiting the NOX2 expression and ROS production. Our results indicate a therapeutic potential of salidroside in the management of oxidative-stress-associated cardiovascular dysfunction.

## 1. Introduction 

Hyperhomocysteinemia is an independent risk factor for various cardiovascular diseases [1–3]. Several Lines of evidence show that homocysteine exerts its adverse effect on endothelial function by increasing oxidative stress and decreasing the activity of nitric oxide synthase [4, 5]. The disrupted vascular permeability, diminished antithrombotic/anticoagulant function, and impaired endothelium-dependent relaxations are the common features of endothelial dysfunction, an initial pathological event leading to the development of atherosclerosis [6, 7].

Salidroside is an active ingredient of the root of *Rhodiola rosea*, a well-known herb used in Tibetan and Chinese medicines to relieve high altitude sickness and to replenish vital energy. The therapeutic action to restore the cardiovascular function has been well documented in Tibetan and Chinese medical literatures. Experimental studies reveal that Rosenroot and salidroside possess, antioxidative, anticancer, antihypoxia, and neuroprotective activities [8–13]. However, as an important herb used to treat cardiovascular diseases, the effect of salidroside on endothelial dysfunction has not been investigated. As homocysteine-induced vascular dysfunction is closely associated with oxidative stress and salidroside is reported to be a strong anti-oxidative agent, we, therefore, hypothesize that salidroside protects against homocysteine-induced endothelial dysfunction. The involvement of endothelial function, endothelial nitric oxide synthase (eNOS) expression, reactive oxygen species (ROS) production, and expression of NADPH oxidase subunits were examined in salidroside-induced vascular benefits. The present study provides new findings in support of salidroside or its containing herbs as a potential medicinal therapy for homocysteine-related cardiovascular diseases.

## 2. Materials and Methods

### 2.1. Animals and Artery Preparation

This study was approved by the Animal Experimentation Ethics Committee, The Chinese University of Hong Kong (CUHK). Male Sprague-Dawley (SD) rats with body weight of 230–250 g were supplied by CUHK laboratory Animal Service Centre. Rats were sacrificed by carbon dioxide inhalation. Aortas were dissected out with surrounding connective tissue removed. Each aorta was cut into several segments, ~4-mm in length in an oxygenated Krebs solution at room temperature.

### 2.2. Protocol

The aortic rings were prepared for function studies, Western blotting and primary culture of endothelial cells for detection of ROS by DHE imaging. Either aortae or aortic endothelial cells were incubated with homocysteine (300 *μ*M) for 45 min to demonstrate the impairment of endothelial function or overproduction of ROS. To test the beneficial effect of salidroside, aortae or endothelial cells were exposed to salidroside (100 *μ*M and 300 *μ*M) for 1 hour prior to the addition of homocysteine. Both tiron (1 mM) and diphenyleneiodonium (DPI, 100 nM) were used as positive controls against homocysteine-induced harmful effects.

### 2.3. Measurement of Isometric Force

Aortic rings were suspended individually in organ baths filled with 10 mL Krebs solution (in mM: NaCl, 119; KCl, 4.7; CaCl_2_, 2.5; MgCl_2_, 1; NaHCO_3_, 25; KH_2_PO_4_, 1.2; D-glucose, 11), oxygenated by 95% O_2_-5% CO_2_, and kept in 37°C. The rings were fixed in one end to a built-in metal hook and connected to a force transducer (AD instrument, USA) on the other end [14]. A basal tension of 2.5 g was maintained for each ring and all rings were allowed to equilibrate for 30 min before the start of the experiment. Rings were first contracted twice by 60 mM KCL solution to ensure the repeatability of contractions. Thereafter, the *α*_1_-adrenoceptor agonist, phenylephrine (0.3 *μ*M), was used to evoke a steady contraction and then relaxed by acetylcholine (ACh, 10 *μ*M) for assessing the presence of functional endothelium. Rings with less than 80% relaxation in response to ACh were discarded. After such initial trials, rings were again contracted by phenylephrine (0.3 *μ*M). Once a stable tension was established, accumulative addition of ACh (3 nM to 3 *μ*M) caused endothelium-dependent relaxations. 

### 2.4. Primary Cell Culture

Primary culture of endothelial cells was prepared from rat aortae under a sterile condition [15]. The aorta was cut open and incubated in PBS solution containing 0.2% of collagenase for enzymatic digestion at 37°C under vigorous shaking. The suspension was centrifuged and cells were resuspended in RPMI Full solution. After 1-hour incubation, the medium was refreshed to remove unattached cells. Thereafter, the cells were placed in an incubator at 37°C with 5% CO_2_ and transferred to a 12-well plate until 70–80% confluence was achieved and incubated for another 1 or 2 days.

### 2.5. Western Blotting

The endothelial cells and aortic tissue were treated according to the protocols used in functional study. They were homogenized in ice-cold RIPA lysis buffer (1 mg/mL leupeptin, 5 mg/mL aprotinin, 100 mg/mL PMSF, 1 mM sodium orthovanadate, 1 mM EGTA, 1 mM EDTA, 1 mM NaF, and 2 mg/mL b-glycerolphosphate). The lysates were centrifuged at 20,000 g for 20 min at 4°C. The supernatants were collected and protein concentration was determined by Lowry method. Samples containing 20 *μ*g of protein were boiled for 10 min with 5%  *β*-mercaptoethanol and separated on a 10% SDS-polyacrylamide gel by electrophoresis. The resolved protein was transferred to an immobilon-P polyvinylidene difluoride membrane (Millipore Corp., Bedford, MA, USA) and blocked with 1% BSA for 20 minutes. Primary antibodies against eNOS, phospho-eNOS (ser1176), NOX2/gp91phox, NOX4 (Abcam), and GAPDH (Ambion, Austin, TX, USA) were used for overnight incubation at 4°C, followed by exposure to horseradish peroxidase-conjugated secondary antibodies (Dako Cytomation, Glostrup, Denmark). An enhanced chemiluminescence detection system (ECL reagents, Amersham Pharmacia Biotech, Buckinghamshire, UK) was used to develop the membranes and a documentation programme (FluorChem, Alpha Innotech Corp., San Leandro, CA, USA) was used for densitometry measurement.

### 2.6. DHE Imaging

Dihydroethidium (DHE) (Molecular Probes, OR, USA) was used to evaluate the amount of oxidant formation [16]. Aortic rings were treated according to the protocols used in functional study and they were incubated in organ baths filled with oxygenated Krebs solution at 37°C and primary endothelial cells were pharmacologically treated. For aortae, frozen sections were prepared in 10 *μ*m thickness using a cryostat microtome (Leica CM1100, Leica Instruments, Germany) and incubated for 10 min in 5 *μ*M DHE at 37°C. For primary endothelial cells, RPMI medium was washed and replaced by NPSS solution and then incubated for 10 min with 5 *μ*M DHE. Fluorescence intensity was measured by confocal microscope (FV1000, Olympus, Tokyo, Japan) with excitation and emission wavelengths of 515 nm and 585 nm, respectively.

### 2.7. ROS Detection by Electronic Paramagnetic Resonance

The level of ROS was measured using the electronic paramagnetic resonance (EPR) technique performed with 1-hydroxy-2,2,6,6-tetramethyl-4-oxo-piperidine hydrochloride (100 *μ*M, Alexis Co., Bingham, UK) as the trapping agent and with diethylenetriaminepentaacetic acid to remove transition metal ions. EPR samples were suspended in PBS solution and placed in 200 *μ*L glass tubes. EMX EPR spectrometer (Bruker, Karlsruhe, Germany) was used to detect the EPR spectra at room temperature [17]. Hypoxanthine-xanthine oxidase (HXXO) and homocysteine were used to show whether they can acutely generate ROS in a cell-free condition.

### 2.8. Chemicals

Salidroside was purchased from Hong Kong Jockey Club Institute of Chinese Medicine Ltd. Phenylephrine, homocysteine, acetylcholine, sodium nitroprusside, hypoxanthine, and DPI were purchased from Sigma while Tiron was purchased from Riedel-de Haën.

### 2.9. Data Analysis

Results are means ± SEM of *n* experiments from different rats. The relaxation was presented as percentage reduction of the evoked tension. Data were analyzed by GraphPad Prism software. The cumulative concentration-response curve was analyzed with a nonlinear curve fitting and the maximal contraction response (*E*_max⁡_) was calculated. Protein expression by Western blotting was normalized to GAPDH and compared with control. Statistical analysis was performed by Students's *t* test or one-way ANOVA followed by Bonferroni post hoc test. A *P* value of less than 0.05 was considered significant.

## 3. Results

### 3.1. Salidroside Improves Homocysteine-Impaired Endothelium-Dependent Relaxation in Rat Aortas

**Figure 1:**
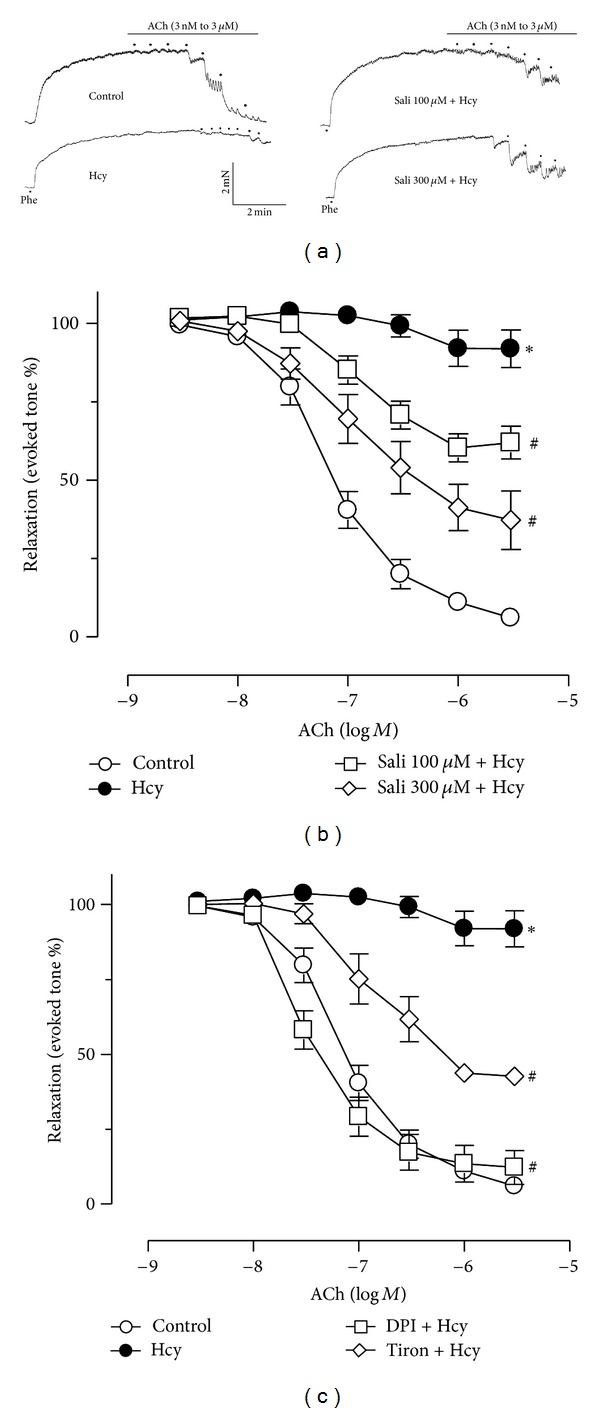
(a) Representative traces showing the impaired endothelium-dependent relaxations (EDR) by 45 min treatment with 300 *μ*M homocysteine (Hcy) and the restoration of EDR by salidroside in rat aortas. The cumulative concentration-response curves for ACh-induced relaxations under treatment with two concentrations of salidroside (b) and with tiron or DPI (c) in Hcy-treated rat aortas. Results are means ± SEM of 6-7 separate experiments. *P < 0.05 versus control; ^#^ *P* < 0.05 versus Hcy.

**Figure 2:**
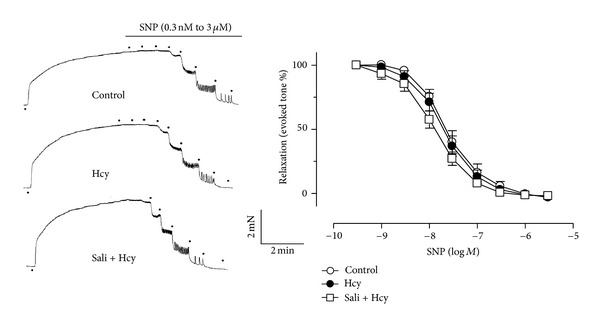
The lack of effect of homocysteine and salidroside on endothelium-independent relaxations of aortas in response to sodium nitroprusside (SNP). Results are means ± SEM of 5 separate experiments.

Treatment of homocysteine (300 *μ*M) for 45 min markedly attenuated ACh-induced endothelium-dependent relaxations (EDRS) when compared with relaxations in control rat aortas (*E*_max⁡_  8.2 ± 5.1 versus 93.3 ± 1.5, *P* < 0.05, Figure 1(b)). Sixty-minute exposure to salidroside (100 *μ*M and 300 *μ*M) prior to the addition of homocysteine partially rescued the impaired EDR in a concentration-dependent manner (Figure 2(b), Table 1). Also, pretreatment with tiron (1 mM) and DPI (100 nM) improved EDR of homocysteine-treated aortas (Figure 1(c)). By contrast, salidroside alone did not modify ACh-induced relaxations (data not shown). L-NAME, the NOS inhibitor at 100 *μ*M, abolished ACh-induced relaxations (data not shown). However, neither homocysteine nor salidroside affected endothelium-independent relaxations in response to sodium nitroprusside (Figure 2).

### 3.2. Salidroside Increases the Level of p-eNOS at ser1176 in Rat Aortic Endothelial Cells

**Figure 3:**
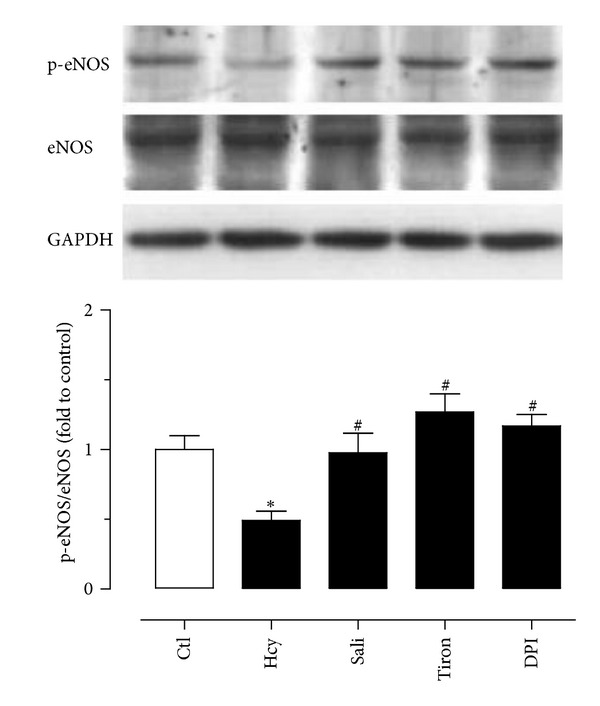
The effect of homocysteine (Hcy) and salidroside (Sali) on the levels of p-eNOS and total eNOS in primary rat aortic endothelial cells. Homocysteine-induced reduction in p-eNOS was reversed by treatment with salidroside, tiron, and DPI. Results are means ± SEM of 4 separate experiments. *P < 0.05 versus control; ^#^ *P* < 0.05 versus Hcy.

Western blot results show that homocysteine (300 *μ*M) reduced the phosphorylation of eNOS at ser1176 compared with control in primary rat aortic endothelial cells. Homocysteine-induced reduction in p-eNOS over total eNOS ratio was reversed by treatment with salidroside (Figure 3). Likewise, tiron and DPI produced the same effect as salidroside in preserving p-eNOS in homocysteine-treated endothelial cells (Figure 3).

### 3.3. Salidroside Reduces the Expression of NOX2 in Rat Aortas

**Figure 4:**
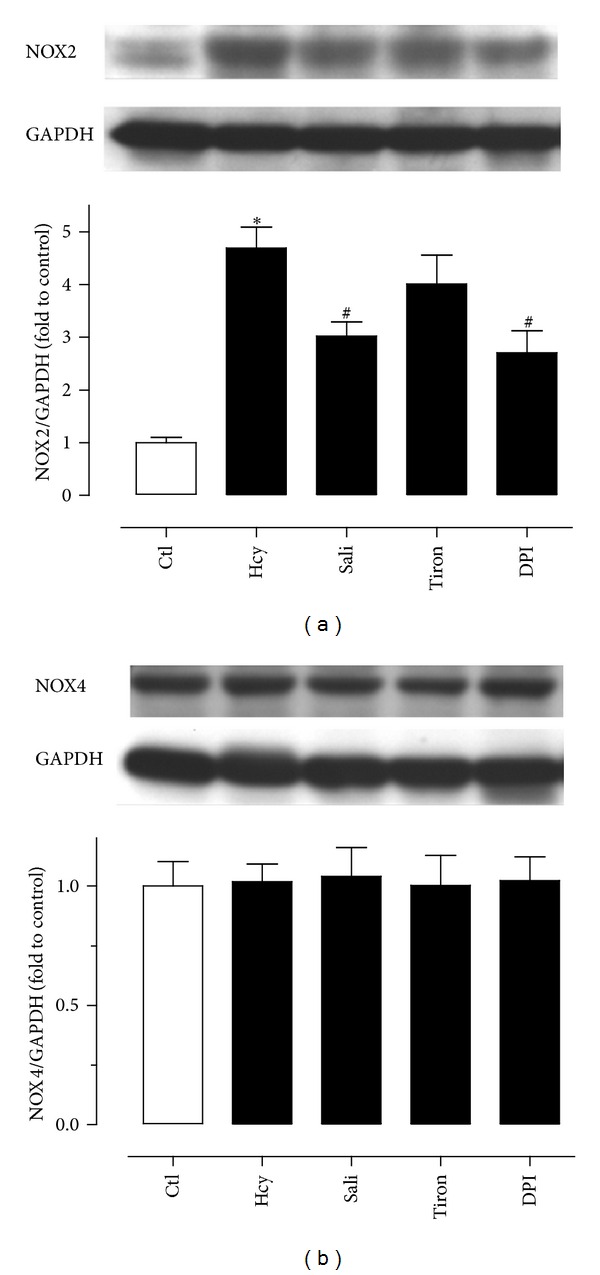
The effect of the expression of NOX2 and NOX4 in rat aortas. (a) Homocysteine (Hcy) increased the NOX2 expression which was partially reversed by salidroside and DPI but not tiron. (b) The NOX4 expression was unchanged in all treatment groups. Results are means ± SEM of 4 separate experiments. *P < 0.05 versus control; ^#^ *P* < 0.05 versus Hcy.

The NOX2 expression was markedly elevated in homocysteine-treated rat aortas when compared with untreated aortas (Figure 4(a)). Pretreatment with salidroside (300 *μ*M) and DPI (100 nM), but not tiron (1 mM), reduced the elevated expression. By contrast, none of these inhibiting agents affected the NOX4 expression (Figure 4(b)).

### 3.4. Salidroside Reduces ROS Production in Rat Aortas and Endothelial Cells

**Figure 5:**
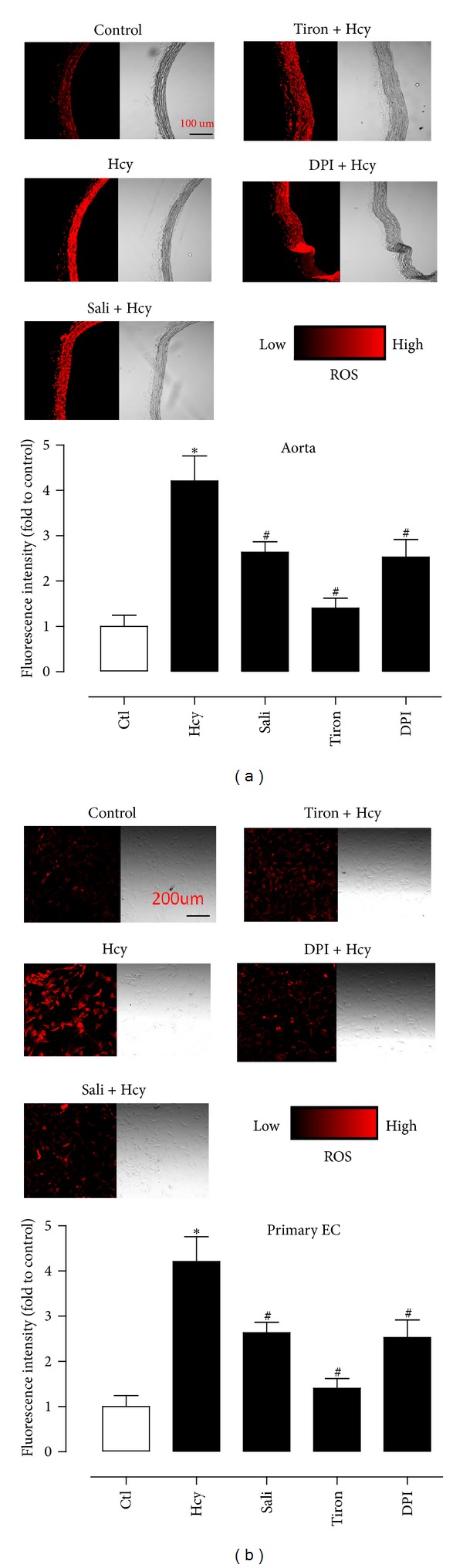
ROS accumulation in aortas (a) and in primary endothelial cells (b) as detected by DHE staining. Homocysteine- (Hcy-) stimulated increase was inhibited by salidroside, tiron, and DPI in ROS in both preparations. Results are means ± SEM of 5 separate experiments. *P < 0.05 versus control; ^#^ *P* < 0.05 versus Hcy.

DHE fluorescence intensity which reflects the ROS level was elevated in both aortas (Figure 5(a)) and primary endothelial cells (Figure 5(b)) in response to 1-hour exposure to homocystein (300 *μ*M) when compared with control cells. Treatment with salidroside, tiron, and DPI significantly reduced the ROS level (Figure 5).

### 3.5. Homocysteine Cannot Acutely Produce ROS

**Figure 6:**
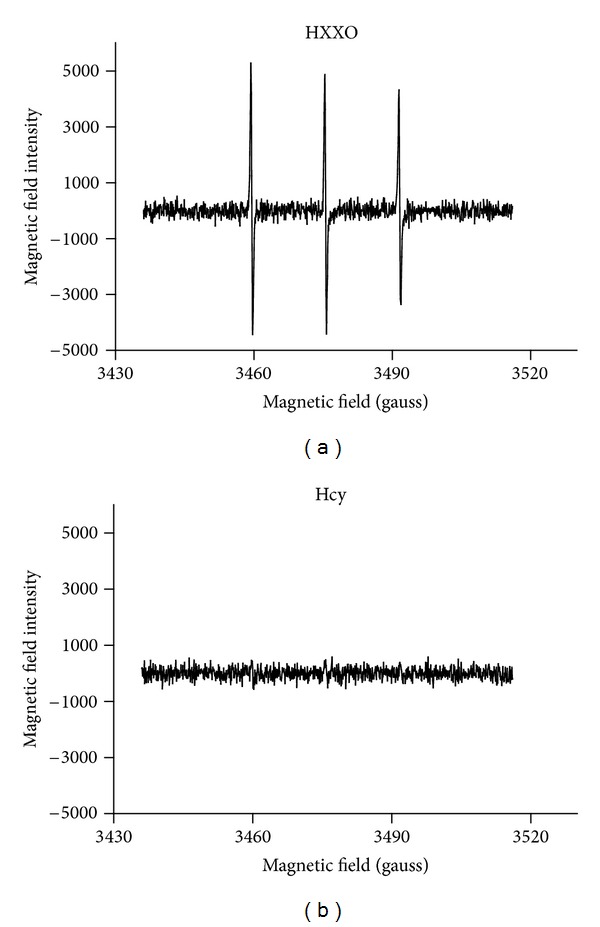
EPR spin trapping showing that hypoxanthine-xanthine oxidase (HXXO) but not 300 *μ*M homocysteine was able to acutely release ROS in a cell-free Krebs solutions reflected by generating EPR spectra. The similar observations were made on four trials.

EPR spin trapping in a cell-free condition shows that hypoxanthine-xanthine oxidase (HXXO) generated EPR spectra while homocysteine at 300 *μ*M did not (Figure 6).

## 4. Discussion

The present study demonstrates that homocysteine impaired endothelium-dependent relaxations in rat aortas and salidroside partially restored the impaired relaxations. This vascular benefits are likely attributed to restoration of diminished NO bioavailability in the presence of homocysteine.

Hyperhomocysteinemia is a known risk factor for developing cardiovascular diseases. Homocysteine injuries endothelial function through an elevated oxidative stress that subsequently reduces the bioavailability of NO [18]. Although our study shows that homocysteine cannot acutely release ROS in a cell-free solution, existing evidence shows that homocysteine promotes the generation of O_2_^−^, H_2_O_2_, and hydroxyl radicals via an autooxidative process of the sulfhydryl group or decreases the intracellular levels of glutathione and glutathione peroxidase which involve ROS elimination [19, 20]. Excessive O_2_^−^ reacts with NO to form peroxynitrite, another highly reactive free radical or uncouples eNOS, ultimately leading to reduced NO function and availability and thus endothelial function. Indeed, our study shows that overnight treatment with homocysteine markedly elevated ROS generation in both endothelium-intact aortas and in primary culture of rat aortic endothelial cells. This ROS overproduction is reversed by tempol, a ROS scavenger or DPI, a tentative inhibitor of NADPH oxidase, indicating a critical role of elevated ROS in homocysteine-induced impairment of endothelium-dependent relaxations.

*Rhodiola rosea* has a long history as a medicinal plant in traditional Tibetan and Chinese medicines. As described in the ancient medical scripts, one of the main functions of *Rhodiola rosea* is to activate vital energy in blood circulation. This herb is effective in reducing high altitude sickness symptoms and fatigue. Among the major bioactive ingredients of the root of *Rhodiola rosea* is salidroside which possesses several potential therapeutic properties including a strong antioxidant activity. Salidroside was reported to restore the impaired mitochondrial function, inhibit accumulation of intracellular ROS, and ameliorate oxidative-stress-induced apoptosis [21, 22]. This compound also protects erythroblasts against oxidative stress by upregulating the expression of antioxidant molecules, glutathione peroxidase, and thioredoxin [8], and it also reverses ischemia-induced cardiomyocyte death through inhibiting ROS overgeneration [23]. Nevertheless, the potential benefit of salidroside against homocysteine-induced endothelial dysfunction was unclear, and the present study provides the first line of experimental evidence to demonstrate that salidroside produces such benefit through curtailing oxidative stress.

In the present study, the alteration of endothelium-dependent relaxations (EDRS) of rat aortas was examined by using ACh which stimulates nitric oxide release from endothelial cells. Homocysteine profoundly attenuated EDR and acute exposure to salidroside significantly restored the impaired relaxations in a concentration-dependent manner. We next examined whether salidroside improved endothelial function through inhibition of oxidative stress in the vascular wall. The present study employed the DHE fluorescence imaging technique to assess changes in the ROS generation and showed that homocysteine markedly elevated ROS generation in both endothelium-intact aortas and in primary culture of rat aortic endothelial cells. Salidroside was potent and effective in reversing the effect of homocysteine in both preparations. Tiron, a ROS scavenger and DPI, a tentative inhibitor of NADPH oxidase serving as positive control were able to abolish the ROS-generating effect of homocysteine. NADPH oxidase is a major source for ROS production. Reducing NADPH oxidase activity could be a cellular mechanism underlying the effect of salidroside in inhibiting homocysteine-induced ROS elevation. We detected the expression of the NADPH oxidase subunits NOX2 and NOX4 and found that the expression of NOX2 but not NOX4 was significantly elevated by homocysteine and this effect was attenuated by salidroside. DPI also inhibited the increased expression of NOX2 while tiron had no effect. Salidroside improves endothelial function at least in a substantial part through curtailing ROS overproduction in the vascular wall. However, the involvement of ROS from other sources such as xanthine oxidase, mitochondrial enzymes, and myeloperoxidase or reduced anti-oxidant capacity cannot be excluded in homocysteine-induced ROS overproduction. Like salidroside, DPI only partially reduced the triggered ROS overproduction. The present study discounts the possibility that homocysteine acutely releases ROS; instead, it is likely to stimulate NOX-2 to generating excessive ROS in the vascular wall and in endothelial cells.

Endothelial nitric oxide synthase (eNOS) catalyzes the biosynthesis of NO in endothelial cells. Phosphorylation of eNOS at ser1176, a serine residue in the reductase domain, activates eNOS while increased ROS directly scavenges NO to lower NO bioavailability [24]. The present study shows that homocysteine markedly reduced the phosphorylation of eNOS which was reversed by salidroside. Tiron and DPI produce similar effects as salidroside against homocysteine, suggesting that increased ROS is likely the main factor to impair endothelial function through reducing eNOS activity in rat arteries.

## 5. Conclusion

In conclusion, salidroside effectively protects rat aortas against homocysteine-induced impairment of endothelium-dependent relaxations through inhibiting NOX2-dependent ROS overproduction and resorting NO bioavailability. The present results may enhance the prospective of using salidroside-containing herbs to ameliorate oxidative stress-associated vascular dysfunction, a common pathological process in hypertension and diabetes.

## Figures and Tables

**Table 1:** ACh-induced relaxations under various pharmacological treatments.

Treatment	pD2	*E*_max⁡_ (%)
ACh relaxation		
Control	7.03 ± 0.06	93.3 ± 1.5
Homocysteine (Hcy)	6.42 ± 0.32	8.2 ± 5.1*
Salidroside 100 *µ*M + Hcy	6.90 ± 0.12	40.4 ± 6.1^#^
Salidroside 300 *µ*M + Hcy	6.97 ± 0.21	62.8 ± 9.3^#^
Tiron + Hcy	7.29 ± 0.10	88.0 ± 3.7^#^
DPI + Hcy	6.72 ± 0.06	52.7 ± 3.9^#^
SNP relaxation		
Control	7.55 ± 0.07	102 ± 0.9
Homocysteine (Hcy)	7.67 ± 0.08	101 ± 0.6
Salidroside 300 *µ*M + Hcy	7.85 ± 0.07	101 ± 1.2

Results are means ± SEM of 5–7 separate experiments. *P < 0.05 versus control and ^#^ *P* < 0.05 versus homocysteine.

## Abbreviations

ACh: Acetylcholine
DHE: Dihydroethidium
DPI: Diphenylene iodonium
EDR: Endothelial-dependent relaxation
eNOS: Endothelial nitric oxide synthase
Hcy: Homocysteine
NO: Nitric oxide
Phe: Phenylephrine
p-eNOS: Phosphorylated endothelial nitric oxide synthase
ROS: Reactive oxygen species
Sali: Salidroside.
